# The risk and nature of flares in juvenile idiopathic arthritis: results from the reACCh-Out cohort

**DOI:** 10.1186/1546-0096-12-S1-O9

**Published:** 2014-09-17

**Authors:** Jaime Guzman, Kiem Oen, Adam M Huber, Gilles Boire, Karen Watanabe Duffy, Roberta Berard, Natalie J Shiff, Deborah M Levy, Elizabeth Stringer, Kimberly Morishita, Rosie Scuccimarri, Lori B Tucker, Rae SM Yeung, Ciaran Duffy

**Affiliations:** 1 U of British Columbia, Vancouver, Canada; 2 U of Manitoba, Winnipeg, Canada; 3 Dalhousie University, Halifax, Canada; 4 U de Sherbrooke, Sherbrooke, Canada; 5 U of Ottawa, Ottawa, Canada; 6 Western University, London, Canada; 7 U of Saskatchewan, Saskatoon, Canada; 8 U of Toronto, Toronto, Canada; 9 McGill University, Montreal, Canada

## Introduction

Accurate description of the risk and nature of flares will help counsel families when JIA is controlled and when considering discontinuing treatment.

## Objectives

To describe the probability and characteristics of flares across JIA categories in an inception cohort of Canadian children treated with a contemporary approach.

## Methods

We studied children diagnosed with JIA between 2005 and 2010 who had at least one visit with inactive disease while being prospectively followed in the Research in Arthritis in Canadian Children emphasizing Outcomes (ReACCh-Out) cohort. They received usual pediatric rheumatology care at 16 Canadian centres. Flare was defined as any recurrence of disease manifestations after attaining inactive disease (no active joints, no extraarticular manifestations and a physician global assessment <10mm). Flares were considered major if they required re-initiation or intensification (a new drug was started) of anti-rheumatic treatment. Risk of flare was calculated with Kaplan-Meier survival methods starting at the time of attainment of inactive disease, and at the time of discontinuing all treatment.

## Results

Of 1492 children recruited in ReACCh-Out, 1128 had at least one visit with inactive disease. Median follow-up was 24 months (IQR 12, 39) after attaining inactive disease. A total of 1,179 flares were observed in 532 patients; 55% of all flares were major flares. The cumulative probability of flare was 25%, 42% and 60% within 6, 12 and 24 months after attaining inactive disease, respectively. By 24 months the risk varied from 49% for systemic JIA to 72% for RF-positive polyarthritis. 625 patients discontinued all treatment. The probability of flares after stopping treatment is shown in the Table (except for RF-positive polyarthritis because only 4 subjects discontinued treatment). Table 1

**Table 1:** Cumulative probability of flare within 6 and 12 months of stopping treatment

JIA category	Subjects stopping treatment / total	Risk within 6 months (%)		Risk within 12 months (%)	
		Any flare	Major flare	Any flare	Major flare

Systemic arthritis	37 / 66	11	3	11	3

Psoriatic arthritis	44 / 75	12	12	15	15

Oligoarthritis	318 / 481	15	12	32	26

Undifferentiated arthritis	60/ 111	24	13	28	18

Enthesitis-related arthritis	77 / 152	29	21	45	33

RF-negative polyarthritis	85 / 209	25	20	45	40

RF-positive polyarthritis	4 / 34	-	-	-	-

## Conclusion

Flares after attaining inactive disease were common in this JIA cohort, and the risk was lowest for systemic JIA and highest for RF-positive polyarthritis. Flares after stopping treatment were uncommon in systemic JIA, but occurred in up to 45% of children within one year in other JIA categories. About half the flares required intensification or re-initiation of treatment.

## Disclosure of interest

None declared.

